# Exercise intervention lowers aberrant serum WISP-1 levels with insulin resistance in breast cancer survivors: a randomized controlled trial

**DOI:** 10.1038/s41598-020-67794-w

**Published:** 2020-07-02

**Authors:** Jae Seung Chang, Tae Ho Kim, In Deok Kong

**Affiliations:** 1 Mitohormesis Research Center, Wonju College of Medicine, Yonsei University, Wonju, Korea; 2 Department of Physiology, Wonju College of Medicine, Yonsei University, Wonju, Korea; 3 Yonsei Institute of Sports Science and Exercise Medicine, Wonju, Korea

**Keywords:** Lifestyle modification, Randomized controlled trials, Breast cancer

## Abstract

Insulin resistance is associated with increased risk for and recurrence of breast cancer. Recently, Wnt1-inducible signaling pathway protein-1 (WISP-1) was reported to impair glucose metabolism and insulin sensitivity. In various cancer tissues, Wnt signaling is upregulated and induces further oncogenic and metastatic activity. However, the effects of exercise on serum levels of WISP-1 and its upstream β-catenin have not been studied in cancer patients. We investigated the effects of exercise training on Wnt signaling and insulin sensitivity in breast cancer survivors (BCS). This single-center trial randomized 46 BCS into either 12-week exercise or control groups (1:1), and included an additional 12 age-matched healthy women. Kinanthropometric parameters, serum Wnt signaling markers, and gluco-lipid profiles were evaluated before and after the intervention. Serum β-catenin and WISP-1 concentrations were significantly higher in BCS than in healthy subjects. There was a positive correlation between β-catenin and WISP-1 levels. Exercise training in BCS significantly reduced body fat and waist circumference and enhanced aerobic and muscular fitness. Exercise decreased β-catenin and WISP-1 levels and improved gluco-lipid profiles. There was a notable correlation between changes in HOMA-IR indexes and serum WISP-1, but not with β-catenin during the exercise intervention. In conclusion, a 12-week community-based exercise intervention resulted in significant reductions in serum β-catenin and WISP-1 levels, accompanied by favorable improvements in body composition, physical fitness, and biochemical parameters in BCS. We also highlight that this is the first report concerning effects of exercise on circulating β-catenin and WISP-1 levels and correlations between WISP-1 and insulin sensitivity, which could be important for determining prognoses for BCS.

## Introduction

Exercise is an important adjunct therapy in the management of cancer for optimal recovery and prevention of treatment-associated complications. Exercise also improves clinical outcomes, including cardiorespiratory and musculoskeletal fitness and recovery from physical and cognitive fatigue^1–3^. Hence, regular exercise in cancer survivors contributes to shortening the time to return-to-work and enhances quality of daily life^4,5^. Although the clinical utility of exercise-oncology has consistently emerged as a critical component of lifestyle modification to improve survival and reduce risk of recurrence^6^, there is less evidence relating exercise to tumorigenesis-related factors, and the biologic mechanisms underlying this association remain unclear.

Wingless and integration site growth factor (Wnt) signaling plays vital roles in numerous cellular processes, including development, differentiation, proliferation, apoptosis, cell motility, and maintenance of the stem cell niche^7^. For instance, Wnt signaling is crucial for breast development during pregnancy and lactation. Despite its physiologic importance, aberrant Wnt signaling is closely linked to tumorigenesis such as the development of breast cancer^8^. Therefore, Wnt-signaling molecules and their regulatory factors are potential biomarkers for cancer diagnosis as well as therapeutic targets^7–10^. Under oncogenic stress, Wnt signaling is triggered and β-catenin accumulates in the cytosol due to inhibition of ubiquitin-proteasomal degradation. The cytosolic β-catenin translocates into the nucleus, binds with T cell factor/lymphoid (TCF) enhancer factor, and acts as a transcriptional cofactor. This complex induces a variety of downstream target genes, including Wnt-induced secreted protein 1 (WISP-1)^10–12^. WISP-1 expression is observed during organ development, wound healing, and tissue repair^13^. WISP-1 expression is abnormally increased in pathologic conditions such as fibrosis and cancers^14^. Consistently, WISP-1 has been suggested to be an oncogene in human breast cancer^15^.

Recently, it has been suggested that WISP-1 is a novel adipokine associated with obesity, hypertriglyceridemia, hyperleptinemia, insulin resistance, and adipose tissue inflammation^16–18^. Given that metabolic disturbances are risk factors for cancer development and poor prognosis, WISP-1 is a candidate pathologic biomarker reflecting both systemic metabolic deterioration and cancer prognosis^19–21^. Furthermore, therapeutic strategies targeting recovery of circulating Wnt-related protein levels are worthwhile to explore for the management of metabolic risk factors in cancer patients.

We hypothesized that regular exercise could be an effective strategy to reduce the expression of Wnt signaling molecules in breast cancer survivors (BCS). The primary aim of this study was, therefore, to examine changes in serum WISP-1 and β-catenin levels elicited by an exercise intervention. Our preliminary observations comprised an age-matched comparison of baseline levels between cancer survivors and healthy persons. For secondary aims, we assessed the effects of the intervention on kinanthropometric (body composition and physical fitness) parameters and blood metabolic profiles, and analyzed their relationships with Wnt signaling indicators.

## Methods

### Study design and ethical approval

The Lifestyles Of Health And Sustainability for Breast Cancer Survivors (LOHAS-BCS) was a single-center, randomized controlled trial. The protocol of this trial was registered at ClinicalTrials.gov (NCT02895178; 09/09/2016). The Medical Ethics Committee of Yonsei University Wonju College of Medicine approved the study protocol (approval number: YWMR-14–0-042), and written informed consent was obtained from all participants. All procedures were conducted in adherence with the Declaration of Helsinki and Consolidated Standards of Reporting Trials (CONSORT).

### Participants

#### Survivors of breast cancer

Women previously diagnosed with histologically confirmed breast cancer were recruited between June 2014 and December 2016 from the hemato-oncology center at Wonju Severance Christian Hospital. Eligibility criteria for inclusion were as follows: (1) diagnosis of stage I‒III breast cancer, (2) completion of all radio- and/or chemotherapy at least 6 months prior to study enrollment, (3) absence of metastatic diseases and other cancers, (4) < 60 min per week of physical activity including resistance exercise in the past 6 months, (5) absence of any musculoskeletal, neurological, metabolic, cardiovascular, and respiratory diseases, and (6) no contraindicated medications and comorbidities that prohibited participation in an exercise program such as the one included in this study.

#### Preliminary data for age-matched healthy volunteers

Twelve age-matched healthy women were included in the initial phase of the study to compare baseline levels of the primary study outcomes between BCS and healthy women without a history of breast cancer.

### Randomization, stratification, and blinding

After baseline assessments, 46 BCS were allocated at a 1:1 ratio to either an exercise intervention group (BCS-Ex) or a control group (BCS-C) using centralized computer-generated random code provided in numbered, sealed, opaque envelopes. Outcome measures were obtained by research assistants blinded to group assignment. The statistician was also unaware of randomized group assignment until completion of the statistical analyses.

### Study intervention

#### Exercise intervention group

The exercise training program followed our previous study protocol^22^, which was designed following the American College of Sports Medicine's guide to exercise and cancer survivorship that recommends optimal exercise intensity, frequency, time, and type^23^. To comprehensively achieve the metabolic and physical benefits from each type of exercise training, we adopt a combined aerobic and resistance exercise regimen in this study. Briefly, each exercise session began with a 10-min warm-up, including stretching and pendulum exercises, for restoring shoulder flexibility. Participants performed a 40-min main exercise program that combined step aerobics using height-adjustable platforms and progressive strength training using various body weights and elastic bands (Thera-Band, Hygenic Co., Akron, OH, USA). Each session ended with a cool-down for 10 min consisting of easy walking and stretching of the engaged muscles. The aerobic exercise was initially performed at a rating of 11–13 on the Borg rating of perceived exertion (RPE) scale and was gradually increased at 4-week intervals until an RPE of 13‒15 was achieved. The strengthening exercise consisted of shoulder presses, black burn exercise, wall push-ups, biceps curls, planks, leg bridges, squats, and calf raises, and was designed to ultimately achieve three sets for each exercise, performing 12‒16 repetitions to volitional fatigue per set. The elastic band resistance was determined using the 2-for-2 rule^24^ and the OMNI-Resistance Exercise Scale (OMNI-RES, 0, extremely easy to 10, extremely hard)^25^. The elastic resistance exercises were performed at an intensity ranging 6–8 on the OMNI-RES which has been noted to correspond to exercise intensity levels ranging 60–80% of the 1 repetition maximum. If a participant could perform two more repetitions in the last set and was reported to be below 6 (somewhat hard) on the OMNI-RES, her elastic band was changed to the next resistance level. Whereas, the body-weight exercises were gradually made more challenging by increasing the number of repetitions and sets conducted for each exercise according to each individual’s improvements. To enhance exercise participant attendance rate, the exercise classes were composed of the same program under the direct supervision of exercise physiologists that were held twice a day on weekdays, and the subjects were encouraged to participate in either of the exercise sessions at least three days weekly for 12 weeks.

#### Control group

Participants in the control group were instructed to maintain their routine occupational and leisure-time physical activity and not to participate any new exercise regimens during the study period. Afterward, participants in the control group who completed both baseline and 12-week follow-up measurements were given the opportunity of participating in the same program as the exercise group.

### Measurements

#### Demographic and clinical characteristics

Demographic characteristics were obtained from personal interviews and self-reported surveys. Clinical characteristics were obtained from electronic medical records.

#### Kinanthropometric profiles

The following measures were obtained at baseline and 12 weeks. Body weight (kg) and height (m) were measured in duplicate and used to calculate body mass index (BMI, kg/m^2^). Waist circumference (cm) was measured at the midpoint between the lower rib margin and iliac crest. Body composition variables involving body fat percentage and skeletal muscle mass were measured using a multi-frequency bioelectrical impedance analysis platform (Inbody 720, Biospace, Seoul, Korea) that has been shown to be an appropriate alternative to dual-energy X-ray absorptiometry^26^. Participants were evaluated for health-related components of physical fitness such as muscular power, strength, endurance, agility, aerobic capacity, and flexibility via the assessments of standing long jump, handgrip strength, sit-ups, 10-m shuttle run, 20-m pacer, and the sit-and-reach test, respectively.

#### Blood collection and biochemical assays

An overnight fasting (≥ 10 h) blood draw was conducted, and separated serum samples were immediately stored at – 80 °C until assayed. WISP-1 concentration was quantified with a chemiluminescence immunoassay (SCG895Hu, Uscn Life Science Inc., Houston, TX, USA). β-catenin concentration was quantified with an enzyme-linked immunosorbent assay (CSB-E08963h, Cusabio Biotech, Newark, DE, USA). Fasting glucose was measured using a hexokinase method (Biosource, Nivelles, Belgium) and fasting insulin was quantified via an electrochemiluminescence immunoassay (Elecsys 2010, Roche, Indianapolis, IN, USA). Lipid profile (triglycerides, total cholesterol and HDL-cholesterol) was analyzed using the enzymatic calorimetric method (Advia 1650, Siemens, Tarrytown, NY, USA), but LDL-cholesterol was based on the calculation [total cholesterol – (1/5 triglycerides) – HDL]. Degree of insulin resistance was calculated using both homeostatic model assessment index 1 (HOMA-IR) and 2 (HOMA2-IR). HOMA-IR was defined as [fasting insulin (μU/ml) × fasting glucose (mg/dl)] / 405^27^. HOMA2-IR was calculated using HOMA Calculator version 2.2.3 (https://www.dtu.ox.ac.uk).

### Statistical analysis

Data are presented as the number of subjects with percentages in parentheses for categorical variables and the mean ± standard deviation for continuous variables. The data were assessed for normality of distribution using Shapiro–Wilk tests. Comparison of categorical variables between groups were conducted by the chi-square test or Fisher’s exact test, and those for continuous variables were analyzed using the independent *t*-test or Mann–Whitney *U* test. To verify the homogeneity of age distribution, we performed comparisons of age between groups using categorized age groups as well as continuous age variables. For this, the participants were categorized into three age groups as follows: (a) 35–44 years, (b) 44–54 years, and (c) 55–64 years. Within-group changes from baseline to 12 weeks were assessed using the paired t-test or Wilcoxon signed-rank non-parametric test. Pearson’s and partial correlations were calculated to assess the relationships between serum levels of Wnt signaling molecules and other clinical parameters. All data were analyzed using SPSS 23.0 software (SPSS, Inc., Chicago, IL, USA). All statistical tests were two-sided, and *P*-values less than 0.05 were considered significant. All graphs were generated using GraphPad Prism 6.0 software (GraphPad Software, Inc., San Diego, CA, USA).

## Results

### Participant characteristics and disposition

**Figure 1:**
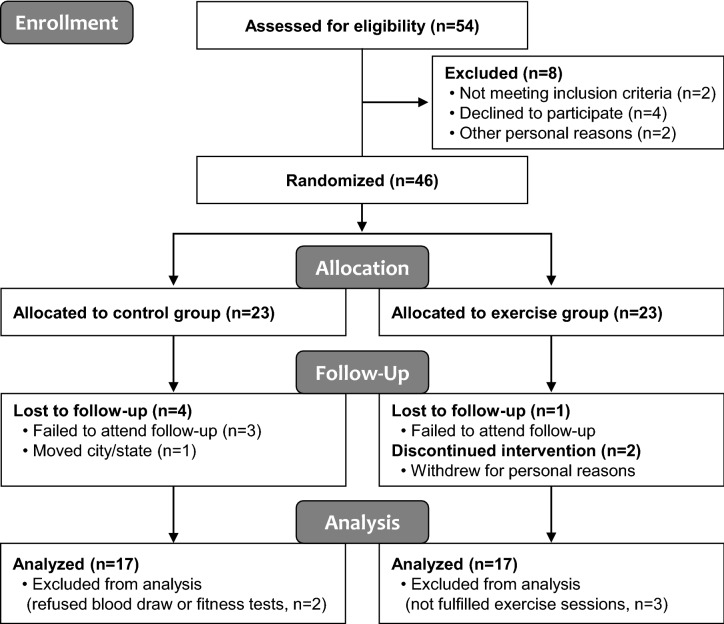
Study flow diagram. This flow diagram shows the study protocol and sequence of events in this study.

A consort diagram of participant flow throughout the study is detailed in Fig. 1. Of the 23 BCS initially assigned to each group, 17 subjects in the BCS-Ex group completed combined exercise training with an average participation rate of 94.8% (range 83.3–100%) for all sessions. We encouraged individuals to participate for at least 3 days weekly, but five were excluded for various reasons (Fig. 1). A total of 34 subjects were eventually included in the final analysis.

### Comparisons between BCS and age-matched healthy women

**Figure 2:**
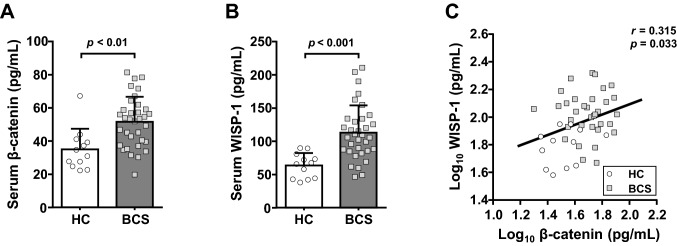
Serum levels of β-catenin and WISP-1 and their intercorrelations among the study participants. Differences in serum β-catenin (**A**) and WISP-1 (**B**) levels between breast cancer survivors (BCS) and age-matched healthy controls (HC). (**C**) Positive correlations between log_10_ β-catenin and log_10_ WISP-1 concentrations in the study participants (n = 46). The Pearson correlation coefficient (*r*) and corresponding regression line are shown. Symbols are individual values and bars represent the means and standard deviations.

Fisher’s exact test and independent *t*-tests indicated no significant differences in age distribution (*p* = 0.504) and mean age (mean ± SD; 50.9 ± 6.3 vs. 50.9 ± 6.8 years, *p* = 0.925) between BCS and healthy controls. In addition, age-matched healthy women had similar demographic characteristics to BCS in this study (Supplementary Table 1). Figure 2 shows the differences in mean serum β-catenin and WISP-1 between BCS and healthy participants. BCS showed significantly higher serum β-catenin levels than age-matched healthy women (mean ± SD; 51.7 ± 15.1 vs. 35.0 ± 12.5 pg/mL, *p* = 0.0014, Mann–Whitney *U* test). Indeed, the baseline serum WISP-1 levels were also significantly higher in BCS than in healthy controls (113.0 ± 41.1 vs. 63.8 ± 18.5 pg/mL, *p* = 0.0002, independent *t*-test). More interestingly, a significant correlation was observed between the log_10_-transformed values of serum β-catenin and WISP-1 concentrations (*r* = 0.315, *p* = 0.033, Pearson’s correlation analysis) (Fig. 2C).

**Table 1:** Changes in kinanthropometric and blood metabolic profiles from baseline to 12 weeks.

Variable	Control (n = 17)			Exercise (n = 17)		
	Baseline	After 12-weeks	*P*-value	Baseline	After 12-weeks	*P*-value
**Anthropometry and body composition**						
Age (years)	50.0 ± 6.1	-		51.4 ± 7.5	-	
Height (m)	155.9 ± 3.6	-		157.0 ± 5.1	-	
Weight (kg)^#^	59.7 ± 10.8	60.1 ± 11.0	0.070	56.0 ± 7.2	55.4 ± 7.5	0.066
BMI (kg/m^2^)	24.6 ± 4.4	24.7 ± 4.4	0.171	22.7 ± 2.6	22.4 ± 2.6	0.064
WC (cm)	81.9 ± 11.4	81.2 ± 13.2	0.371	78.7 ± 7.0	75.3 ± 6.8	0.005
Affected-arm circumference (cm)	29.86 ± 3.78	30.14 ± 3.72	0.095	28.24 ± 1.86	27.77 ± 1.67	0.013
Unaffected-arm circumference (cm)	29.64 ± 3.59	29.94 ± 3.70	0.120	28.09 ± 1.89	27.84 ± 1.73	0.082
Body fat (%)	33.2 ± 7.3	34.5 ± 7.0	0.009	33.0 ± 5.4	31.9 ± 4.9	0.031
SkMM/BMI^#^	0.878 ± 0.117	0.841 ± 0.151	0.005	0.886 ± 0.094	0.901 ± 0.091	0.049
**Health-related physical fitness**						
HGS (kg)^#^	24.2 ± 3.8	24.8 ± 4.6	0.423	22.6 ± 4.3	25.2 ± 2.6	0.001
Standing long jump (cm)^#^	114.7 ± 35.3	118.4 ± 37.0	0.394	122.6 ± 17.4	119.9 ± 20.3	0.394
Sit-up (n/30 sec)	14.7 ± 9.2	15.1 ± 9.8	0.609	11.6 ± 8.7	18.7 ± 9.8	0.001
10 m shuttle run (sec)^#^	16.3 ± 3.9	17.0 ± 4.3	0.192	14.8 ± 1.1	14.6 ± 1.1	0.113
20 m pacer (n)^#^	12.1 ± 6.6	13.9 ± 6.5	0.832	11.6 ± 5.4	14.4 ± 7.7	0.039
Sit-and-reach (cm)	14.2 ± 6.4	13.0 ± 7.1	0.279	8.9 ± 9.7	11.7 ± 8.0	0.006
**Blood variables**						
Triglyceride (mg/dL)^#^	116.6 ± 65.0	131.9 ± 91.7	0.583	110.0 ± 49.9	102.1 ± 50.5	0.099
Total cholesterol (mg/dL)	181.4 ± 33.2	178.2 ± 36.5	0.547	192.6 ± 28.1	178.8 ± 27.9	0.023
HDL-cholesterol (mg/dL)	53.6 ± 17.9	54.3 ± 20.3	0.767	55.7 ± 7.7	58.4 ± 9.7	0.259
LDL-cholesterol (mg/dL)	104.8 ± 30.0	96.7 ± 30.2	0.116	114.3 ± 31.0	99.5 ± 26.7	0.018
Fasting glucose (mg/dL)	95.3 ± 21.3	98.1 ± 23.9	0.184	88.6 ± 14.8	92.2 ± 6.8	0.358
Fasting insulin(µIU)^#^	13.1 ± 10.2	12.5 ± 11.0	0.492	10.9 ± 6.8	8.1 ± 3.4	0.035
HOMA1-IR^#^	3.11 ± 2.69	3.06 ± 2.92	0.653	2.47 ± 1.46	1.83 ± 0.80	0.025
HOMA2-IR^#^	1.68 ± 1.31	1.62 ± 1.41	0.463	1.40 ± 0.84	1.05 ± 0.44	0.035
HOMA1-β^#^	154.1 ± 94.1	140.2 ± 105.1	0.193	151.3 ± 130.2	105.4 ± 47.8	0.058
HOMA2-β^#^	120.3 ± 50.4	111.0 ± 56.6	0.210	114.1 ± 60.2	94.3 ± 28.9	0.068

*BMI* body mass index; WC, waist circumference, *SkMM/BMI* skeletal muscle mass to BMI ratio, *HGS* handgrip strength, *HDL* high density lipoprotein, *LDL* low density lipoprotein.

*P*-values obtained by Student’s paired *t-*test or ^#^Wilcoxon signed-rank test.

Values are the mean ± SD.

### Effect of exercise intervention on study outcomes

Baseline demographic and clinical characteristics of the randomized BCS are described in Supplementary Table 2. There were no baseline differences between BCS-C and BCS-Ex groups for anthropometric, body composition, or physical fitness variables (Supplementary Table 3). In addition, no statistically significant differences were found between the groups for any of the medical history and blood biochemical parameters.

**Table 2:** Relationships of serum WISP-1 levels with fasting insulin and HOMA-IR in breast cancer survivors.

	Baseline				After 12-weeks				Delta (after 12 weeks—baseline)			
	Simple correlation		Partial correlation†		Simple correlation		Partial correlation‡		Simple correlation		Partial correlation^¶^	
	r*	*P-value*	*r*	*P-value*	r*	*P-value*	*r*	*P-value*	r*	*P-value*	*r*	*P-value*
Fasting glucose (mg/dL)	0.017	0.925	-0.117	0.524	0.464	0.006	0.349	0.052	0.044	0.806	0.098	0.595
Fasting insulin(µIU)	0.655	< 0.001	0.487	0.005	0.668	< 0.001	0.485	0.005	0.452	0.007	0.408	0.020
HOMA-IR	0.597	< 0.001	0.391	0.027	0.690	< 0.001	0.526	0.002	0.438	0.010	0.398	0.024
HOMA2-IR	0.640	< 0.001	0.463	0.008	0.673	< 0.001	0.495	0.004	0.442	0.009	0.401	0.023

*Pearson's correlation coefficient.

Adjusted by age and BMI at baseline† or after 12-weeks‡.

^¶^Adjusted by age and delta BMI (after 12-weeks ‒ baseline).

#### Kinanthropometric parameters

Table 1 shows changes in the outcome variables from baseline to post-intervention. BCS-Ex, but not BCS-C, displayed significant reductions of 1.1% in body fat percentage and 4.3% in waist circumference, with an increase of 1.7% in relative skeletal muscle mass index. Likewise, there were also significant enhancements in physical fitness variables only in the BCS-Ex group. Muscular strength and aerobic capacity were significantly increased through exercise training by 11.5% and 26.3%, respectively as measured by a handheld dynamometer and a 20-m pacer test. Muscular endurance as assessed by the sit-up test and flexibility by the sit-and-reach test also increased by 61.2% and 31.5%. However, muscular power and agility remained unchanged after 12 weeks.

#### Blood gluco-lipid profiles

The exercise intervention led to noteworthy improvements in biochemical and serological variables related to metabolic status (Table 1). Of the serum lipid components, TC and LDL-C were significantly decreased by 7.2% and 12.9% in the BCS-Ex group after 12 weeks, respectively, but not in the BCS-C group. Fasting insulin concentration was significantly decreased by 25.9% after exercise training, whereas the change in fasting glucose level was statistically non-significant. More interestingly, HOMA-IR and HOMA2-IR indexes in the BCS-Ex group noticeably declined by 29.5% and 25%, respectively. In contrast, these biochemical variables did not change in the BCS-C group after 12 weeks.

#### Primary outcomes: Changes in serum WISP-1 and β-catenin after exercise intervention

**Figure 3:**
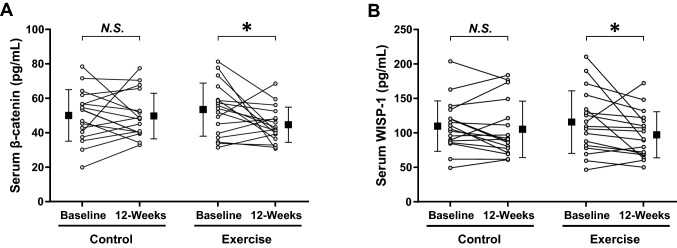
Individual and mean changes in (**A**) β-catenin and (**B**) WISP-1 serum levels elicited by 12-weeks of exercise training in breast cancer survivors. Big symbols with bars are the means and standard deviations of individual values (circles). *p < 0.05 by Wilcoxon signed-rank tests; N.S., not statistically significant.

Individual and mean changes in serum β-catenin and WISP-1 levels are shown in Fig. 3. A 12-week exercise intervention elicited noteworthy reductions in β-catenin (baseline vs. after 12-week, mean ± S.D., 53.4 ± 15.4 vs. 44.5 ± 10.2 pg/mL, *p* = 0.046, Wilcoxon signed-rank test) and WISP-1 (115.9 ± 45.9 vs. 97.4 ± 33.7 pg/mL, *p* = 0.031, Wilcoxon signed-rank test) levels in the serum of BCS-Ex group, whereas the changes in the BCS-C group were not significant (β-catenin: 49.9 ± 15.0 vs. 49.7 ± 13.2 pg/mL, *p* = 0.938; WISP-1, 110.1 ± 36.8 vs. 105.4 ± 41.1 pg/mL, *p* = 0.407, Wilcoxon signed-rank tests).

### Associations of WISP-1 with insulin and HOMA-IR in BCS

Table 2 shows the relationships of circulating WISP-1 levels with insulin concentrations and HOMA-IR indexes in BCS. Pearson’s correlation analyses showed that serum WISP-1 levels at baseline were positively correlated with fasting insulin (*r* = 0.655, *p* < 0.0001), HOMA-IR (*r* = 0.597, *p* = 0.0002), and HOMA2-IR (*r* = 0.640, *p* < 0.0001). Likewise, such correlations were also consistently observed at 12-week follow-up. Partial correlation analyses showed that these positive associations remained significant even after adjusting for age and BMI. To determine the relationships between intervention-induced changes in WISP-1 and anthropometric and metabolic parameters, the coefficient correlation analyses were carried out using the delta values of each variable (at 12-week follow-up – baseline). Interestingly, Pearson’s correlation analyses revealed positive correlations of delta WISP-1 with delta insulin (*r* = 0.452, *p* = 0.0072), delta HOMA-IR (*r* = 0.438, *p* = 0.0096), and delta HOMA2-IR (*r* = 0.442, *p* = 0.0088). Moreover, partial correlation analyses demonstrated that the positive associations between delta values remained significant even after adjustments for age and delta BMI.

## Discussion

This is the first randomized controlled study to examine the effects of exercise on two biomarkers associated with breast cancer. We found clear evidence for the beneficial effects of exercise on clinical parameters and both biomarkers. Along with an increased proportion of cancer survivors, non-pharmacologic supportive healthcare strategies counteract the adverse effects of cancer treatments and comorbidities. As one of these strategies, lifestyle modification, has received great interest because of its practical efficiency in the continuing care and rehabilitation of chronic patients^28^. Exercise, particularly combined aerobic-strength exercise for cancer patients, is known to have beneficial consequences. This exercise regimen improves body composition, reduces stiffness of the affected arm and shoulder, and increases cardiorespiratory and musculoskeletal fitness in BCS^6^. Arm edema is a common symptom in patients and in survivors after breast cancer surgery, resulting in substantial functional limitation of the affected region^29^. Our data indicate that affected-arm circumferences were significantly reduced in the BCS-Ex group, implying the alleviation of edematous changes (Supplementary Table 3). To assess the effectiveness of the exercise intervention applied in this study, we evaluated health-related parameters including body composition, physical fitness, and biochemical markers. A 12-week exercise intervention led to successful enhancement of muscular and aerobic fitness and flexibility with body composition improvement resulting from reduced body fat percentage and increased relative muscle mass. In addition, the exercise intervention elicited beneficial changes in biochemical markers such as decreased fasting insulin, total- and LDL-cholesterol levels, and ameliorated insulin resistance.

While accumulating evidence has demonstrated good outcomes due to physical exercise among cancer patients, the molecular mechanisms underlying its beneficial role has not been investigated extensively. We revealed that the favorable effects of exercise intervention were accompanied by reductions in circulating levels of tumorigenic Wnt signaling molecules such as β-catenin and WISP-1. To our knowledge, this is the first report showing alterations in circulating Wnt downstream molecules due to a clinical or lifestyle intervention. Previously, we observed that exercise training reduced serum levels of DKK1, a negative regulator of Wnt signaling^22^. In fact, DKK1 expression can be directly up-regulated by Wnt-induced β-catenin/TCF activation, but it inhibits the Wnt signaling pathway, thereby constituting a negative feedback loop^30^. It has been demonstrated that high serum levels of DKK1 are associated with poor prognosis among breast cancer patients, since it might reflect an activated status of tumorigenic Wnt/β-catenin signaling.

Cytosolic β-catenin is a key mediator between activation of Wnt signaling and subsequent increase of WISP-1 expression^31,32^. We found that serum β-catenin as well as WISP-1 levels were higher in BCS compared to healthy controls. We also detected a significant positive correlation between β-catenin and WISP-1 in serum samples from our subjects. Constitutive activation and mutation of β-catenin play causative roles in mammary gland tumorigenesis, which is suggested to be a prognostic marker for breast cancer^32^. Moreover, enrichment of β-catenin in the cytosol and nucleus is more frequently observed in basal-like invasive breast cancers^33^. In patients with hepatocellular carcinoma, serum β-catenin levels were higher than those in hepatitis or healthy controls. Our results also suggest the possibility that circulating β-catenin may be used as a diagnostic and/or prognostic biomarker in breast cancer. Further research would be valuable to explore this hypothesis.

Previous histological evidence has shown that WISP-1 expression is associated with more advanced features including breast cancer stage, tumor size, and lymph node metastasis^34^. Transcript profiling demonstrated high WISP-1 expression in a variety of tumors, including breast cancer^14,35^. Meanwhile, WISP-1 is a promising diagnostic biomarker for prostate cancer because it is highly detected not only in tissue biopsies but also in patient serum^36^. Notably, WISP-1 has recently been identified as a pro-inflammatory adipokine, and its circulating levels are associated with metabolic risk factors and systemic inflammatory markers. In insulin-sensitive tissue, WISP-1 decreases the phosphorylation of insulin receptor and protein kinase B (akt). As a consequence, glycogen synthesis is reduced in myotubes, and gluconeogenic genes are upregulated in hepatocytes^17^. Accordingly, we observed significant correlations of serum WISP-1 level with fasting insulin, HOMA-IR, waist circumference, BMI, and body fat percentage in a cross-sectional analysis (Supplementary Fig. 1). In addition, WISP-1 level was inversely correlated with physical fitness parameters (Supplementary Fig. 2). To elucidate the role of exercise in WISP-1 and insulin sensitivity, we compared these parameters before and after the exercise intervention. As expected, exercise training reduced serum WISP-1 and increased insulin sensitivity. Interestingly, the decrement in WISP-1 after exercise showed significant correlations with changes in HOMA-IR, even after adjusting for age and delta BMI. However, changes in β-catenin and HOMA-IR were not correlated. These results imply that WISP-1 and insulin sensitivity have a close connection that is different from that between β-catenin and HOMA-IR.

It is noteworthy that the prevalence of breast cancer and its progression are closely related to insulin resistance and metabolic diseases^37–39^. Therefore, BCS prognosis could be sensitively influenced by exercise-induced metabolic interventions. Our findings unveiled a positive correlation between changes in WISP-1 and insulin resistance and physical exercise in BCS. The beneficial effect on insulin sensitivity due to exercise may not originate from a single factor. Nevertheless, we suggest that reduced WISP-1 partly contributes to improvements in insulin sensitivity and cancer prognosis. In this regard, in vitro and in vivo experimental studies have recently demonstrated that WISP-1 not only promotes growth of human breast cancer cells by downregulating a tumor suppressor (N-myc downstream-regulated gene 1) and cell-cycle inhibitors (cyclin-dependent kinase inhibitors 1 and 1B), but that it also encourages metastatic potential by stimulating epithelial to mesenchymal transition traits. Thus, WISP-1 is a potential therapeutic target as an independent tumorigenic risk factor for human breast cancer^15^.

There are several limitations regarding the applications of this trial. The main limitation of the study was the involvement of a single medical center (and the correspondingly small sample size), which may constrain the generalizability of our findings. Other limitations were the duration of intervention and the absence of follow-ups during the detraining period. These shortcomings limit our ability to comment on the sustainability of the observed beneficial changes by exercise over a longer time horizon. Further multi-center studies evaluating detraining effects on biomarkers with long-term follow-up could provide more valuable insights regarding supportive care for cancer survivors. On the other hand, since this study investigated the effect of a combined aerobic and resistance exercise training regimen on circulating Wnt-related protein levels, the question yet remains which type of exercise is more effective or whether these two exercise types work together synergistically as well as separately. To develop exercise guidelines to improve cancer patient prognosis, further research is warranted to determine which exercise regimen has a larger effect on the aberrant circulating levels of tumorigenic risk factors, such as WISP-1.

According to a review of the individual data, contrary to the overall trends, some participants showed increases in WISP-1 (3 subjects) and β-catenin (5 subjects) after the exercise intervention. Intriguingly, it was also found that all of those with a rise in serum WISP-1 level among the exercised participants also had increases in triglyceride and insulin levels, and HOMA-IR indexes as well. However, these features were not consistently observed in those with a rise in serum β-catenin level. Although the study data are insufficient to interpret the mechanistic causes of the non-responses to exercise training observed in those few cases, the simultaneous increases in circulating WISP-1 level and insulin resistance further suggest the close association between these factors’ changes with respect to each other. Recently, it has been suggested that heterogeneity of response to exercise exists and could be influenced by various endogenous (e.g. age, race, and genetics) and exogenous (e.g., modes of exercise, nutritional status, and medications) factors on an individual basis^40^. Differing volumes, doses, and intensities of exercise training with other lifestyle modifications or medications may be exploited to achieve beneficial responses in patients observed to be non-responders to exercise interventions. In addition, accumulating epidemiological evidence indicates that the impact of exercise on prognostic outcomes may appear to differ across breast tumor subtypes^41,42^, but it is currently unclear whether certain tumor subpopulations are more responsive to exercise in breast cancer survivors^43^. Moreover, the underlying mechanisms that drive variation in metabolic and hormonal responses to exercise are still unknown, especially in cancer patients. Unfortunately, however, we did not obtain the clinicopathological profiles of the study participants because the initial study design did not consider a comparison of the study outcomes according to intrinsic molecular subtypes of breast cancer. Thus, not being able to investigate the relationship between breast cancer subtypes and the circulating level of Wnt-related molecules is another limitation of the present study. In order to establish the efficacy of exercise regimens as an adjunct strategy to reduce the risks of poor prognosis in patients with breast and other solid tumors, it will be valuable for future studies to identify the association between heterogeneity in response to exercise and cancer outcomes on the basis of tumor histopathologic and molecular features as well as changes in potential biomarkers.

In conclusion, we observed that WISP-1 and β-catenin, key mediators in Wnt signaling, decreased alongside improvement in metabolic parameters during and after exercise training. In particular, WISP-1 is known to have close connections to insulin signaling and tumorigenesis. This is the first prospective observational study to document changes in serum Wnt signal molecules due to an exercise intervention. We suggest that WISP-1 and its regulators are potential candidates as diagnostic/prognostic biomarkers and therapeutic targets in BCS.

## Supplementary information

Supplementary file1 (PDF 732 kb)

Supplementary file2 (PDF 273 kb)

Supplementary file3 (PDF 145 kb)
