# Supplementary material for: Exercise intervention lowers aberrant serum WISP-1 levels with insulin resistance in breast cancer survivors: a randomized controlled trial
Source: Sci Rep. 2020 Jul 2;10:10898. doi: 10.1038/s41598-020-67794-w (PMC7331642; doi:10.1038/s41598-020-67794-w)
Supplement: Supplementary file 1 — Supplementary file1 (PDF 732 kb) [file 41598_2020_67794_MOESM1_ESM.pdf]

Supplementary information

Article title: Exercise intervention lowers aberrant serum WISP-1 levels with insulin resistance in breast cancer survivors: a randomized controlled trial

Journal: Scientific Reports

Author: Jae Seung Chang, Ph.D., Tae Ho Kim, Ph.D., In Deok Kong, M.D., Ph.D.

Corresponding author: In Deok Kong, Yonsei University Wonju College of Medicine. E-mail: kong@yonsei.ac.kr

Supplementary Table 1. Demographic characteristics of the study participants.

Variables	Age-matched healthy women (n = 12)	Breast cancer survivors (n =34)	*P*-value
Age (years)	50.9±6.3	50.9±6.8	0.925
Age group, n (%)[¶]			
35 – 44	2 (16.6)	4 (11.8)	0.504
45 – 54	5 (41.7)	21 (61.7)	
55 – 64	5 (41.7)	9 (26.5)	
Anthropometry and body composition			
Height (cm)	155.2±5.1	156.5±4.4	0.317
Weight (kg)*	55.5±6.8	57.8±9.3	0.617
WC (cm)	78.2±5.6	79.8±9.6	0.485
BMI (kg/m^2)*	23.0±2.3	23.6±3.7	0.920
Body fat (%)	29.5±5.6	33.1±6.3	0.086
SkMM (kg)	21.5±5.6	20.6±2.2	0.348
SkMM/BMI	0.934±0.145	0.881±0.105	0.185
Health-related physical fitness			
HGS (kg)*	26.4±5.1	23.5±4.1	0.157
Standing long jump (cm)	130.1±15.7	122.7±18.5	0.225
Sit-up (n/30sec)	20.2±11.0	13.3±8.9	0.035
10 meter shuttle run (sec)*	14.8±1.5	15.4±2.7	0.305
20 meter pacer (n)*	15.1±5.4	12.3±5.9	0.123
Sit-and-reach (cm)	15.5±5.7	11.5±8.6	0.142
Blood variables			
Triglyceride (mg/dL)	131.5±38.0	111.6±54.2	0.231
Total cholesterol (mg/dL)*	179.8±24.2	187.4±29.1	0.476
HDL-cholesterol (mg/dL)	55.7±13.8	55.1±30.4	0.909
LDL-cholesterol (mg/dL)	105.4±23.6	108.6±30.4	0.745
Fasting glucose (mg/dL)*	89.5±16.0	93.4±15.6	0.471

BMI, body mass index; WC, waist circumference; SkMM, skeletal muscle mass; SkMM/BMI, SkMM to BMI ratio; HGS, handgrip strength; HDL, high density lipoprotein; LDL, low density lipoprotein.

Continuous variables compared using independent *t*-test or *Mann-Whitney *U* test.

Nominal variables compared using [¶]Fisher's exact test.

Values are the mean ± SD or n (%).

Supplementary Table 2. Baseline demographic and clinical characteristics of breast cancer survivors randomly assigned to exercise intervention and control groups.

Variable	Control (n =17)	Exercise (n =17)	*P*-value
Age range (years)			
Age group, n (%)			
35 – 44	1 (5.9)	3 (17.6)	0.20
45 – 54	13 (76.5)	8 (47.1)	
55 – 64	3 (17.6)	6 (35.3)	
Marital status			
Married/living as married	17 (100)	17 (100)	1.00
Education status			
Middle school or less	4 (23.5)	3 (17.6)	0.59
High school	7 (41.2)	10 (58.8)	
College or higher	6 (35.3)	4 (11.8)	
Smoking history			
Never-smoker	17 (100)	17 (100)	1.00
Menopausal status			
Postmenopausal	13 (76.5)	14 (82.4)	0.67
Type of surgery			
Mastectomy	2 (11.8)	2 (11.8)	1.00
Lumpectomy	15 (88.2)	15 (88.2)	
Treatment			
Chemotherapy	2 (11.8)	-	0.34
Radiation	1 (5.9)	1 (5.9)	
Chemotherapy and radiation	14 (84.3)	16 (94.1)	
Comorbidities			
Dyslipidemia	3 (17.6)	3 (17.6)	1.00
Type 2 diabetes mellitus	2 (11.7)	1 (5.9)	0.54
Hypertension	5 (29.4)	4 (23.5)	0.70
Cardiovascular diseases	1 (5.9)	-	0.31
Thyroid disease	4 (23.5)	2 (11.8)	0.37
Osteoporosis	1 (5.9)	1 (5.9)	1.00
Arthritis	1 (5.9)	-	0.31

P-values obtained by the chi-square test or Fisher's exact test.
Values are the number of study participants with the percentage in parentheses.

Supplementary Table 3. Baseline kinanthropometric and blood metabolic profiles of breast cancer survivors randomly assigned to exercise intervention and control groups.

Variable	Control (n = 17)	Exercise (n =17)	*P*-value
Anthropometry and body composition			
Age (year)	50.0±6.1	51.4±7.5	0.553
Height (m)	155.9±3.6	157.0±5.1	0.459
Weight (kg)¶	59.7±10.8	56.0±7.2	0.335
BMI (kg/m^2)	24.6±4.4	22.7±2.6	0.137
WC (cm)	81.9±11.4	78.7±7.0	0.530
Affected-arm circumference (cm)	29.86±3.78	28.24±1.86	0.126
Unaffected-arm circumference (cm)	29.64±3.59	28.09±1.89	0.131
Body fat (%)	33.2±7.3	33.0±5.4	0.903
SkMM/BMI¶	0.878±0.117	0.886±0.094	0.744
Health-related physical fitness			
HGS (kg)¶	24.2±3.8	22.6±4.3	0.168
Standing long jump (cm)¶	114.7±35.3	122.6±17.4	0.918
Sit-up (n/30sec)	14.7±9.2	11.6±8.7	0.262
10 meter shuttle run (sec)¶	16.3±3.9	14.8±1.1	0.148
20 meter pacer (n)¶	12.1±6.6	11.6±5.4	0.378
Sit-and-reach (cm)	14.2±6.4	8.9±9.7	0.070
Blood variables			
Triglyceride (mg/dL)¶	116.6±65.0	110.0±49.9	0.692
Total cholesterol (mg/dL)	181.4±33.2	192.6±28.1	0.302
HDL-cholesterol (mg/dL)	53.6±17.9	55.7±7.7	0.831
LDL-cholesterol (mg/dL)	104.8±30.0	114.3±31.0	0.152
Fasting glucose (mg/dL)	95.3±21.3	88.6±14.8	0.892
Fasting insulin(μIU)¶	13.1±10.2	10.9± 6.8	0.783
HOMA1-IR¶	3.11±2.69	2.47±1.46	0.931
HOMA2-IR¶	1.68±1.31	1.40±0.84	0.877
HOMA1-β¶	154.1±94.1	151.3±130.2	0.718
HOMA2-β¶	120.3±50.4	114.1±60.2	0.718

BMI, body mass index; WC, waist circumference; SkMM/BMI, skeletal muscle mass to BMI ratio; HGS, handgrip strength; HDL, high density lipoprotein; LDL, low density lipoprotein.
P-values obtained by independent *t*-test or ¶Mann-Whitney test.
Values are the mean ± SD.

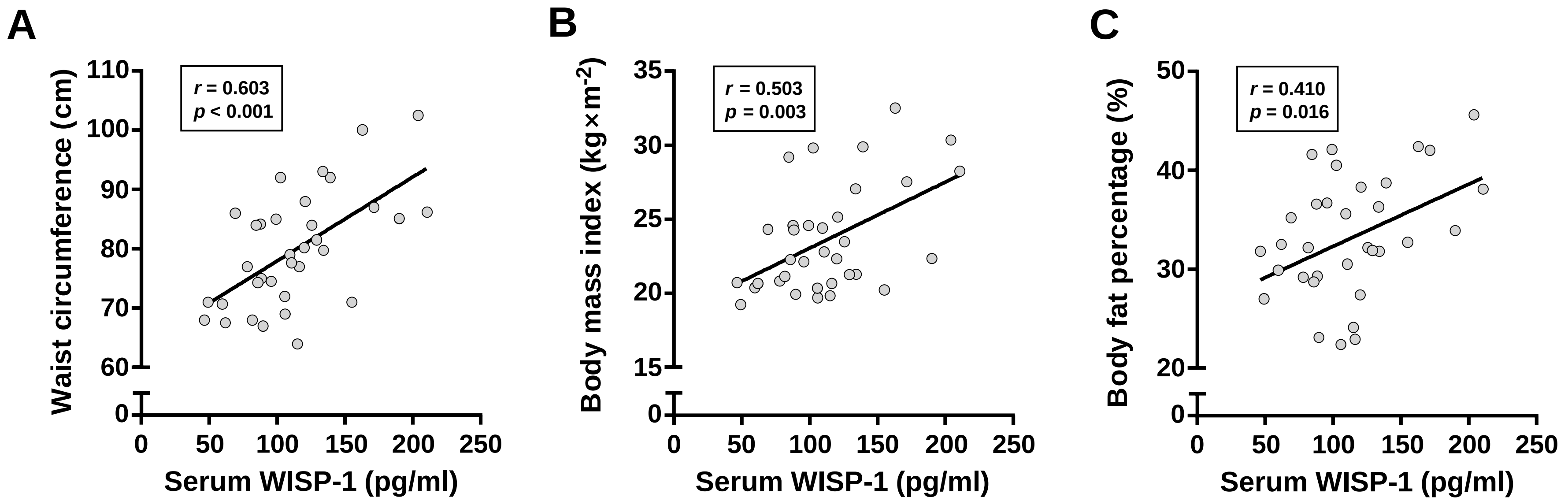

Supplementary Figure 1. Correlations between serum WISP-1 levels and obesity indicators. Relationships between serum WISP-1 concentrations and waist circumference (A), BMI (B) and body fat percentage (C) in breast cancer survivors. r, Pearson's correlation coefficient

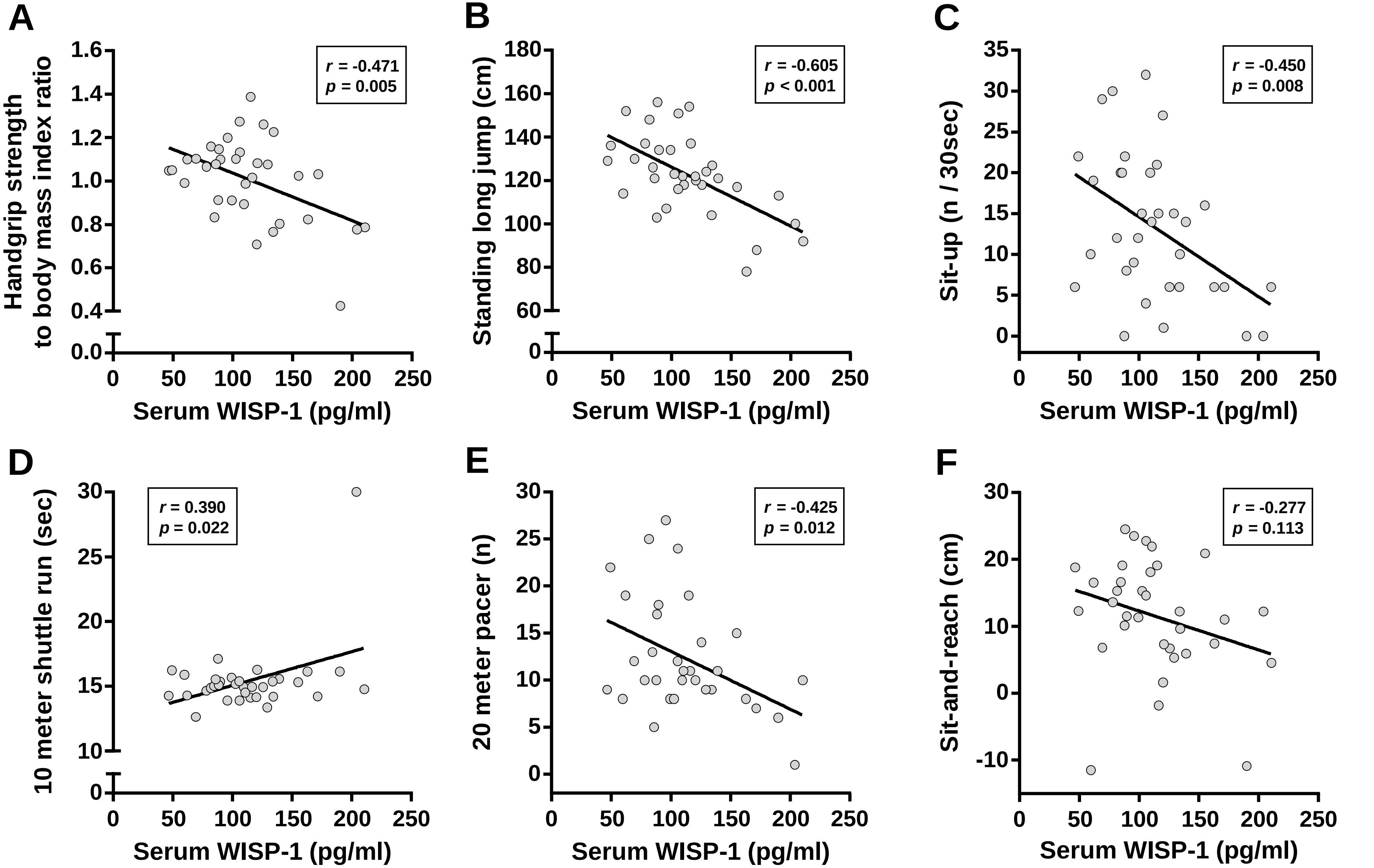

Supplementary Figure 2. Correlations between serum WISP-1 levels and physical fitness parameters. Relationships between serum WISP-1 concentrations and muscular strength (A), muscular power (B), muscular endurance (C), agility (D), aerobic capacity (E) and flexibility (F) in breast cancer survivors. r, Pearson's correlation coefficient.
