# Supplementary material for: Exercise intervention lowers aberrant serum WISP-1 levels with insulin resistance in breast cancer survivors: a randomized controlled trial
Source: Sci Rep. 2020 Jul 2;10:10898. doi: 10.1038/s41598-020-67794-w (PMC7331642; doi:10.1038/s41598-020-67794-w)
Supplement: Supplementary file 2 — Supplementary file2 (PDF 273 kb) [file 41598_2020_67794_MOESM2_ESM.pdf]

YONSEI UNIVERSITY
WONJU COLLEGE OF MEDICINE

Department of Physiology and Hematology-Oncology

Clinical Study Protocol

Effects of exercise training on WNT signaling-related biomarkers in breast cancer survivors: a randomized controlled trial

STUDY PROTOCOL SUMMARY

**Title**	**Effects of exercise training on WNT signaling-related biomarkers in breast cancer survivors: a randomized controlled trial.**
**Introduction**	Despite the general benefits known to patients with cancer, the effects of exercise on the initiation and progression of the tumor itself remain unclear. Abnormal Wnt signaling is one of the major tumorigenesis-related signaling pathways. Particularly, aberrant Wnt signaling is closely linked to tumorigenesis such as the development of breast cancer. Therefore, Wnt-signaling molecules and their regulatory factors are potential biomarkers for cancer diagnosis as well as therapeutic targets. Under oncogenic stress, Wnt signaling is triggered and β-catenin accumulates in the cytosol due to inhibition of ubiquitin-proteasomal degradation. The cytosolic β-catenin translocates into the nucleus, binds with T cell factor/lymphoid (TCF) enhancer factor and acts as a transcriptional cofactor. This complex induces a variety of downstream target genes, including Wnt-induced secreted protein 1 (WISP-1). WISP-1 expression is abnormally increased in pathologic conditions such as fibrosis and cancers. Recent studies have demonstrated that WISP-1 not only promotes growth of human breast cancer cells by downregulating tumor suppressor genes and cell-cycle inhibitors, but it also encourages metastatic potential by stimulating epithelial to mesenchymal transition traits. Thus, WISP-1 is a potential therapeutic target as an independent tumorigenic risk factor for human breast cancer.
**Design**	LOHAS-BCS study is a two-armed 1:1 randomized controlled study in breast cancer survivors.
**Target population**	Breast cancer survivors who have completed adjuvant or palliative systemic therapy after surgical treatment.
**Number of centers and setting**	Single center. Department of Physiology and Department of Hematology-Oncology, Yonsei University Wonju College of Medicine
**Objectives**	To investigate the effect of an exercise-based intervention on circulating levels of Wnt signaling molecules and metabolic disease biomarkers, physical function/fitness and body composition.
**Selection criteria**	**Inclusion Criteria:**<br>▪ Have diagnosed as a stage of I-III breast cancer<br>▪ Have undergone lumpectomy or mastectomy<br>▪ Have completed neoadjuvant/adjuvant chemotherapy and able to initiate exercise program<br>▪ Nonsmokers (i.e., not smoking during previous 12 months)<br>▪ Able to provide physician clearance to participate in exercise program for 12 weeks<br><br>**Exclusion Criteria:**<br>▪ History of chronic disease including diabetes, uncontrolled hypertension or thyroid disease<br>▪ Weight reduction >= 10% within past 6 months<br>▪ Metastatic disease<br>▪ Participate more than 60 minutes of exercise per week in the past 6 months<br>▪ Cardiovascular, respiratory or musculoskeletal disease or joint problems that preclude moderate physical activity

**Study arms**	Participants will be randomized 1:1 to either an exercise intervention group or a control group.<br>Participants in the intervention group will receive standard care and a 12-week exercise-based intervention comprised of:<br>1) Supervised group exercise training at the exercise medicine center three times a week. Each session will last about 60 minutes.<br>2) Home-based exercise will be encouraged at least 1 day a week during the intervention period<br><br>Participants in the control group will receive standard care.
**Baseline and endpoint data**	**Primary outcomes**<br>▪ <u>Oncogenic Wnt signaling related biomarkers</u>: serum levels of WISP-1 and β-catenin<br>▪ <u>Health-related physical fitness</u>: handgrip strength (muscular strength), sit-up (muscular endurance), standing long jump (muscular power), multi-stage 20-meters PACER test (cardiorespiratory fitness), 10-meter shuttle run test (agility), sit and reach (flexibility)<br>▪ <u>Risk parameters of metabolic diseases</u>: Waist circumference, BMI (anthropometry), body fat and segmental lean mass (body composition), fasting glucose and insulin levels, total-, HDL- and LDL-cholesterols, homeostasis model assessment indices (blood gluco-lipid profiles)<br><br>**Secondary outcomes**<br>▪ Adipokines: serum levels of leptin and adiponectin<br>▪ Inflammatory-related cytokines: serum levels of IL-1β, IL-10, IL-11 and TNF-α<br>▪ Cancer-related biomarkers: serum levels of osteoprotegerin, osteopontin and growth differentiation factor-15
**Number of participants / sample size**	A 25%-point difference in serum Wnt signaling molecule levels within each group are considered to be significant in this study. In order to detect the rate of change between baseline and 12-week intervention in both groups, and obtain a type 1 error rate of 5% and a power of 90%, a sample size of 17 subjects per study arm are needed. To account for an expected dropout rate of ~30%, we decided to increase this number to a group size of 25. Thus, a total of 50 breast cancer survivors will be included in the study. The minimum number of subjects for adequate study power was calculated with a sample size calculator.
**Statistical analysis**	Feasibility measures (acceptability, adherence, and adverse events etc.) are reported as numbers and percentages. Reasons for declining participation are analyzed descriptively with numbers and percentages of subjects declining for various reasons. Descriptive statistics are calculated to identify the means and standard deviations (SD) or standard errors of the mean (SEM). After Shapiro-Wilk normality test and variances analysis, parametric paired t-test or non-parametric Wilcoxon signed-rank test are used to compare the levels of health-related fitness, body composition, and serum biomarkers between before and after training, as appropriate. Pearson's and partial correlations are calculated to assess the relationships between serum levels of Wnt signaling molecules and other clinical parameters. All data are analyzed using SPSS 22.0 software (SPSS, Inc., Chicago, IL, USA). All statistical tests are two-sided and *P*-values less than 0.05 are considered significant.

**Potential risks and disadvantages**	There are a few potential risks and disadvantages for participants in the intervention group, including exercise injuries and discomfort in performing the exercise program. However, the risks and disadvantages are considered to be limited. Two experienced exercise specialists will supervise all exercise sessions at the community-based exercise medicine center operated by medical college and center. If a participant shows any signs of being physically unwell during and after an exercise session, the person will be provided a primary medical care in the hospital setting, and appropriate actions will be taken. For safety reasons, subjects who have fever (>38.5 °C), infectious disease or musculoskeletal injury are not allowed to participate in the training session. If a participant reports any discomfort or incidence of injury during the intervention period, safety and further program continuation for the participant will be discussed in the research group, and in consultation with the participant and the responsible physician. Investigators and responsible oncologists have the rights to exclude a participant from the study at any given time, if it is considered unsafe for the participant to continue. Data on adverse events will be collected systematically every week, and participants will further be informed to contact the primary investigator immediately if injuries or other adverse events related to the intervention program are required.
**Scientific statement**	The LOHAS-BCS study is expected to involve low risk of adverse events and discomfort for participants involved. It is the project group's assessment that it is necessary to achieve the current research project to gain important knowledge about the effect and feasibility of exercise-based intervention as a rehabilitative approach to cancer survivors. The project group presumes that the exercise intervention of LOHAS-BCS will maintain or increase physical fitness levels and tolerance to oncological treatment, reduce symptoms and side effect, and improve psychological well-being and quality of life in the patients with breast cancer. Therefore, it is estimated that the expected benefits of the exercise intervention exceeds the potential risks and disadvantages for participants involved in the project.

RATIONALE

Patients and survivors of breast cancer present impaired physical fitness and various complications including acute and chronic pain, severe fatigue, limited range of motion, and bone loss attributable to anticancer treatments[1,2]. Therefore, regular exercise during and following cancer treatments has been recommended to enhance physical capabilities and relieve severity of side effects, leading to an improved quality of life[2-4]. Although the clinical utility of exercise-oncology has consistently emerged as a critical component of lifestyle modification to improve survival and reduce risk of recurrence[4], there is less evidence relating exercise to tumorigenesis-related factors and the biologic mechanisms underlying this association remain unclear. Despite the general benefits known to patients with cancer, the effects of exercise on the initiation and progression of the tumor itself remain unclear.

Wingless and integration site growth factor (Wnt) signaling plays vital roles in numerous cellular processes, including development, differentiation, proliferation, apoptosis, cell motility, and maintenance of the stem cell niche[5]. For instance, Wnt signaling is crucial for breast development during pregnancy and lactation. Despite its physiologic importance, abnormal Wnt signaling is one of the major tumorigenesis-related signaling pathways[6]. Particularly, aberrant Wnt signaling is closely linked to tumorigenesis such as the development of breast cancer[7]. Therefore, Wnt-signaling molecules and their regulatory factors are potential biomarkers for cancer diagnosis as well as therapeutic targets[7-9].

Under oncogenic stress, Wnt signaling is triggered and β-catenin accumulates in the cytosol due to inhibition of ubiquitin-proteasomal degradation. The cytosolic β-catenin translocates into the nucleus, binds with T cell factor/lymphoid (TCF) enhancer factor and acts as a transcriptional cofactor. This complex induces a variety of downstream target genes, including Wnt-induced secreted protein 1 (WISP-1)[9-11]. WISP-1 expression is observed during organ development, wound healing, and tissue repair[12]. However, WISP-1 expression is abnormally increased in pathologic conditions such as fibrosis and cancers[13]. Recently, an in vitro and in vivo study has demonstrated that WISP-1 not only promotes growth of human breast cancer cells by downregulating tumor suppressor genes and cell-cycle inhibitors, but it also encourages metastatic potential by stimulating epithelial to mesenchymal transition traits[14]. Thus, WISP-1 is a potential therapeutic target as an independent tumorigenic risk factor for human breast cancer.

On the other hand, it has been suggested that WISP-1 is a novel adipokine associated with obesity, hypertriglyceridemia, hyperleptinemia, insulin resistance and adipose tissue inflammation[15-17]. Notably, as a consequence, glycogen synthesis is reduced in myotube, and gluconeogenic genes are upregulated in hepatocytes[16]. Given that metabolic disturbances are risk factors for cancer development and poor prognosis, WISP-1 is a candidate pathologic biomarker reflecting both systemic metabolic deterioration and cancer prognosis[18-20]. Furthermore, therapeutic strategies targeting recovery of circulating Wnt-related protein levels are worthwhile to explore for the management of metabolic risk factors in cancer patients.

OBJECTIVES

We hypothesize that regular exercise could be an effective strategy to reduce expression of Wnt signaling molecules in breast cancer survivors. The primary aim of this study is to compare the changes in serum WISP-1 and β-catenin levels elicited by exercise intervention. As a preliminary observation to this, an age-matched comparison in those baseline levels between cancer survivors and healthy persons will be conducted. As secondary aims, we will assess the effects of the intervention on kinanthropometric (body composition and physical fitness) parameters and blood gluco-lipid metabolic profile and analyze their relationship with Wnt signaling indicators.

METHODOLOGY

Study design

Randomization The Lifestyles Of Health And Sustainability for Breast Cancer Survivors (LOHAS-BCS) is a single-center, two-armed randomized controlled trial. Eligible participants are randomly assigned to either an exercise intervention group or a control group using sealed, computer generated random numbers with the allocation ratio of 1 to 1.

Study procedures Demographic characteristics are collected from personal interviews and self-reported surveys. Clinical characteristics are obtained from electrical medical records. Measurements are conducted at baseline and 12-week intervention, which include anthropometry, body composition, health–related fitness levels, and blood sample collection.

Blinding Four research staff members who are unaware of group assignment performed all outcome assessments. The statistician is unaware of treatment allocation until completion of the statistical analyses. Participants are not blind to their assignment but are unaware of main outcome measures, and are instructed to avoid mentioning anything regarding their study experience to the assessors.

Study participants

Survivors of breast cancer, who visited the hemato-oncology center of Wonju Severance Christian Hospital, are recruited between June 2014 and December 2016. Treatment history and menstrual status are obtained from the clinical reports of the hemato-oncology center with the consent of participants. Written informed consent will be obtained from all participants included in the study. They are eligible to participate in this study if they met the following inclusion criteria without exclusion criteria, with medical clearance from their oncologist.

1. **Inclusion Criteria**:
    1) Have diagnosed as a stage of I-III breast cancer
    2) Have undergone lumpectomy or mastectomy
    3) Have completed neoadjuvant/adjuvant chemotherapy and able to initiate Exercise program
    4) Nonsmokers (i.e., not smoking during previous 12 months)
    5) Able to provide physician clearance to participate in exercise program for 12 weeks

2. **Exclusion Criteria**:
    1) History of chronic disease including diabetes, uncontrolled hypertension or thyroid disease
    2) Weight reduction >= 10% within past 6 months
    3) Metastatic disease
    4) Participate more than 60 minutes of exercise per week in the past 6 months
    5) Cardiovascular, respiratory or musculoskeletal disease or joint problems that preclude moderate physical activity

Interventions

Exercise group The exercise training program is designed following the American College of Sports Medicine cancer guidelines (exercise intensity, frequency, time, and type), and are performed at least three times per week for total 12 weeks, under the direct supervision of exercise physiologists. Exercise training is conducted with individual's ratings of perceived exertion intensities within 11–13 that are gradually and moderately increased at 4-week intervals until reaching a rating of 13–15. Each session begins with a warm-up consisting whole body stretching and flexibility exercises for shoulder muscles stiffness such as finger climbing, shoulder glides (inferior, anterior, and posterior), and pendulum exercises for 10 min. The exercise program incorporates step aerobics on 17-cm (6.7-inch) platforms for 20 min followed by the strength training using body weight consisting of shoulder press, black burn exercise, wall push-up, biceps curl-up, plank exercise, leg bridge, squat, and calf raise for 20 min. At the end of the session, subjects perform cool-down involving easy walking and stretching exercises for 10 min (Table 1). Subjects in the exercise group are encouraged to participate in a home-based exercise session at least 30 minutes once and more weekly for 12 weeks.

Control group Participants in the control group are instructed to maintain their routine physical activities and not to participate any new exercise programs during the 12-weeks study period. Afterwards, the subjects who complete both pre and post-test received an opportunity to participate in the same exercise program that the intervention group has performed.

Table 1. Supervised and group-based exercise intervention program for 12-weeks

Contents	Work out		Frequency	Time	Intensity
**Warm-up**	Shoulder static stretching<br>Dynamic stretching			10 min	RPE 10
**Main exercise**	Step aerobics	Step jacks / side jacks<br>March step/jacks<br>Step back + knee up<br>Side-to-side steps<br>Side leg swings<br>Cross step arm extension,<br>Alternating steps back, etc.	10~20 rep.	20 min	1 ~ 4 wks, RPE 11~13<br><br>5 ~ 12 wks, RPE 13~15<br><br>9 ~ 12 wks, RPE 15
	Upper body	Shoulder press<br>Arm/triceps curls	12~16 rep. x 2~3 set	20 min	OMNI-Resistance Exercise Scale 6~8
	Trunk/core	Crunch<br>Plank<br>Bridge<br>Leg raise<br>Dead lift			
	Lower body	Squat<br>Lunge<br>Hamstring, kickback			
**Cool-down**	Shoulder static stretching<br>Dynamic stretching			10 min	RPE 10

RPE, ratings of perceived exertion

Observations

Primary outcomes The aim of the study is to assess and understand the effects of an exercise program on health-related physical fitness and biomarkers involving cancer-related molecules. For this reasons we will evaluate:

1. Changes of cancer-related biomarkers such as serum levels of Wnt1-inducible signaling pathway protein-1 (WISP-1) and its upstream β-catenin.
2. Components of health-related physical fitness including cardiorespiratory fitness, muscular exercise capacity and flexibility.
3. Risk parameters of metabolic diseases including anthropometric and body composition variables, and blood gluco-lipid profiles.

.

Outcome measures

1. Wnt signaling-related biomarkers

Blood samples are drawn from the antecubital vein and collected in serum separation tubes. The tubes are centrifuged at 3000 rpm (1,000 ×g) for 10 min, and serum samples are collected and immediately stored at -80°C until analysis. Commercially available enzyme-linked immune sorbent assay (ELISA) kits are used to measure the changes of serum levels of Wnt signaling-related molecules such as WISP-1 and

β-catenin according to the manufacturer's protocol.

1) WISP-1
   - Product code, SCG895Hu
   - Manufacturer, USCN Life Science Inc.
   - Minimal detectable density (sensitivity), 0.97 pg/mL
   - Standard curve range, 2.74 - 2,000 pg/mL
   - Intra-assay, CV<10%; inter-assay, CV<12%
   - Test method: double-antibody sandwich; detection wavelength, 450 nm

2) β-catenin
   - Product code, CSB-E08963h
   - Manufacturer, CUSABIO Technology
   - Minimal detectable density (sensitivity), 3.9pg/mL
   - Standard curve range, 15.6 - 1,000 pg/ml
   - Intra-assay, CV<8%; inter-assay, CV<10%
   - Test method: double-antibody sandwich; detection wavelength, 450 nm

2. Health-related physical fitness

1) Muscular strength is assessed by the maximum voluntary strength of handgrip (kg). Grip strength is measured 4 times, twice each of the dominant and non-dominant hands, and the mean and maximum values is recorded to the nearest 0.1 kg (TKK-5401, Takei, Japan).

2) Muscular endurance is assessed using sit-up test. The maximum repetitions for 30 seconds are recorded. To assure the starting position, the participants lie on their back with knees flexed, feet on the floor with hands on the opposite shoulders. Arm contact with the chest must be maintained. The chin should remain tucked on the chest. The sit-ups are completed when the elbows touch the thighs. To complete the sit-up, the participants returns to the down position until the mid-back makes contact with the testing surface (BS-SU, InBody Co., Korea).

3) Muscular power is assessed using standing long jump test. The maximum horizontal distance of two trials is recorded to the nearest 1 centimeter.

4) Aerobic capacity is assessed using Progressive Aerobic Cardiovascular Endurance Run (multi-stage

20-meters PACER test). Participants are instructed to run back and forth between two lines, 20-meters apart for the time allowed at the sound of a tape-recorded beep. The test score is recorded as the number of shuttles completed on pace, and only one trial is permitted. Before the actual test, all participants perform a short pilot trial run to help them understand how to pace themselves. During the test, all participants are encouraged with positive feedback such as “good job”, “keep it going” etc. When the participants are unable to make it to the marks on two consecutive beeps, the test are terminated and instructed to walk around to cool-down.

5) Agility is assessed using 10-meters agility shuttle run test. Two parallel lines were drawn on the floor 10 m apart. Participants run twice back and forth as fast as possible, covering a total distance of 20 m, while picking up a designated piece of wood placed at the furthest point. Time recording is stopped when the participants crossed the end line with one foot. The lower value of two trials is recorded to the nearest 0.01 second.

6) Flexibility is assessed using sit and reach test with a flexometer (BS-FF, InBody Co., Korea). The test is performed by sitting with the feet barefoot straight against the vertical plane of the measuring instrument. Then with both hands gathered and knees fully upright, the upper body bends forward and pushes the meter as far forward as possible. The point where the fingertip stops at about 2 seconds was measured and the high value was recorded to the nearest 0.1 centimeter.

3. Risk parameters of metabolic diseases

1) Anthropometric parameters

- Body weight and height are measured to the nearest 0.1 kg and 0.1 cm using an automatic scale after taking off the shoes and maintaining an upright posture (BSM370, BioSpace Inc., Korea).
- Waist circumference is measured at the midpoint between the lower rib margin and the iliac crest using a non-stretch clear measuring tape.
- Body mass index is calculated as weight (kg) divided by height squared (m^2)

2) Body composition parameters

- Body fat and segmental lean mass (arms, legs and trunk) are measured by a bio-impedance analyzer after removing all accessories and metals (Inbody720, BioSpace Inc., Korea).
- Percentage body fat is calculated as body fat mass (kg) divided by weight (kg).
- Skeletal muscle mass is calculated from the sum of appendicular (arms + legs) and trunk lean masses (kg).

3) Blood gluco-lipid profiles

- Fasting glucose is analyzed using a hexokinase enzymatic method (Biosource, Nivelles, Belgium) with an assay reagent (Glucose HK Gen.3 kit, Germany)
- Fasting insulin is analyzed by an electrochemiluminescence immunoassay (Elecsys 2010, Roche, Indianapolis, IN, USA) with an assay reagent (Elecsys Insulin, Roche Diagnostic, Germany).
- Total cholesterol, triglycerides and high-density lipoprotein are analyzed using an enzymatic colorimetric method (Advia 1650, Siemens, Tarrytown, USA) with the Cholesterol and Triglycerides Liquiform (Labtest Diagnostica, Lagoa Santa, Brazil) and D-HDL kits (Siemens Diagnostics, Tarrytown, USA), respectively.
- The homeostasis model assessment (HOMA) indices are used to evaluate insulin resistance (HOMA-IR) and pancreatic β-cell function (HOMA-β). HOMA-IR is calculated as *fasting insulin (µU/ml) × fasting glucose (mg/dl) /405*. HOMA-β is calculated as *360 × fasting insulin concentration (µU/ml) / [fasting glucose (mg/dl) – 63]*. Additionally, we confirm our findings using the HOMA2 approach. HOMA2-IR and HOMA2-β data are calculated with a HOMA2 calculator released by the Diabetes Trials Unit, University of Oxford: HOMA Calculator. This calculator is available at: http://www.dtu.ox.ac.uk/homacalculator/index.php.

Secondary outcomes The serum levels of adipokines, inflammatory and cancer-related cytokines are measured as secondary outcome measures. After which, we will examine the relationship between the changes of each biomarker including cancer-related molecules elicited by an exercise program.

Outcome measures

1. Serum levels of adipokines.

The serum concentration of leptin and adiponectin are measured by commercial ELISA kits (R&D systems).

- Leptin, minimal detectable density, 7.8 pg/ml; standard curve range, 15.6 - 1,000 ng/ml
- Adiponectin, minimal detectable density, 0.891 ng/ml; standard curve range, 3.9 - 250 ng/ml

2. Serum levels of inflammatory-related cytokines

The serum concentration of IL-1 beta, IL-10, IL-11 and TNF-alpha are measured using commercial luminex multiplexed cytokine assay panels (R&D systems).

- IL-1 beta, minimal detectable density, 0.8 pg/ml; standard curve range, 17.8 - 4,320 pg/ml
- IL-10, minimal detectable density, 1.6 pg/ml; standard curve range, 13.7 - 3,340 pg/ml
- IL-11, minimal detectable density, 24.7 pg/ml; standard curve range, 0.5 - 125.4 ng/ml
- TNF-alpha, minimal detectable density, 1.2 pg/ml; standard curve range, 14 - 3,410 pg/ml

3. Serum levels of cancer-related molecules.

The serum concentrations of osteoprotegerin, osteopontin, and growth differentiation factor 15 (GDF-15) are measured by commercial luminex multiplexed cytokine assay panels (R&D systems).

- Osteoprotegerin, minimal detectable density, 3.62 pg/ml; standard curve range, 81.4 - 19,770 pg/ml
- Osteopontin, minimal detectable density, 413 pg/ml; standard curve range, 3.4 - 826.9 ng/ml
- GDF-15, minimal detectable density, 1.2 pg/ml; standard curve range, 34 - 8,270 pg/ml

4. Serum high-sensitivity C-reactive protein is measured by electrochemiluminescence immunoassay (Roche cobas 8000-e801, Roche Diagnostics, Tokyo, Japan).

Sample size

No prior studies have investigated the minimal clinically important difference or change in the circulating levels of WISP-1 and the β-catenin focusing on breast cancer patients. However, according to a previous study focusing on patients with gestational diabetes mellitus, their serum WISP-1 levels were significantly higher by 20% compared to that of healthy pregnant women[21]. In addition, according to another calorie restriction study focusing on obese patients, the rate of clinically meaningful reduction in circulating WISP-1 level was around 27.5% in obese women[22]. Based on results from prior studies, a 25%-point difference in the serum Wnt signaling molecule levels within each group are considered to be significant in this study. In order to detect the rate of change between baseline and 12-week intervention in both groups, and to obtain a type 1 error rate of 5% and a power of 90%, a sample size of 17 subjects per study arm are needed. To account for an expected dropout rate of ~30%, we decided to increase this number to a group size of 25. Thus, a total of 50 breast cancer survivors will be included in the study. The minimum number of subjects for adequate study power was calculated with a sample size calculator released by ClinCalc LLC. This calculator is available at https://clincalc.com/stats /samplesize.aspx. The formula used for power and sample size calculations is as follows:

The anticipated mean and standard deviation of group #1 = 2104 ± 472 pg/mL[22]

The anticipated percent difference in outcomes of group #2 compared to group #1 = 25%

$k = n_2 / n_1 = 1$

$n_1 = (\sigma_1^2 + \sigma_2^2 / K) \times (z_{1-\alpha/2} + z_{1-\beta})^2 / \Delta^2$

$n_1 = (472^2 + 472^2 / 1) \times (1.96 + 1.28)^2 / 526^2$

$n_1 = 17$

$n_2 = K \times n_1 = 17$

$\Delta = |\mu_2 - \mu_1|$ = absolute difference between two means

σ_1, σ_2 = variance of mean #1 and #2

n_1 = sample size for group #1

n_2 = sample size for group #2

α = probability of type I error (set as 0.05)

1 - β = probability of type II error (set as 90%)

z = critical Z value for a given α or β

k = ratio of sample size for group #2 to group #1

Data analysis

Feasibility measures (acceptability, adherence, and adverse events etc.) are reported as numbers and percentages. Reasons for declining participation are analyzed descriptively with numbers and percentages of subjects declining for various reasons. Descriptive statistics are calculated to identify the means and standard deviations (SD) or standard errors of the mean (SEM). After Shapiro-Wilk normality test and variances analysis, parametric paired t-test or non-parametric Wilcoxon signed-rank test are used to compare the levels of health-related fitness, body composition, and serum biomarkers between before and after training, as appropriate. Pearson's and partial correlations are calculated to assess the relationships between serum levels of Wnt signaling molecules and other clinical parameters. All data are analyzed using SPSS 22.0 software (SPSS, Inc., Chicago, IL, USA). All statistical tests are two-sided and *P*-values less than 0.05 are considered significant.

ETHICAL CONSIDERATIONS

Approval by ethics review committees

All procedures were reviewed and approved by the Medical Ethics Committee of Yonsei University Wonju College of Medicine, Korea (YWMR-14-0-042). Written informed consent is obtained from all individual participants included in the study. All procedures are conducted in adherence with the Declaration of Helsinki and Consolidated Standards of Reporting Trials.

Potential risks and disadvantages

There are a few potential risks and disadvantages for participants in the intervention group, including exercise injuries and discomfort in performing the exercise program. However, the risks and disadvantages are considered to be limited. Two experienced exercise specialists will supervise all exercise sessions at the community-based exercise medicine center operated by medical college and center. If a participant shows any signs of being physically unwell during and after an exercise session, the person will be provided a primary medical care in the hospital setting, and appropriate actions will be taken. For safety reasons, subjects who have fever (>38.5 °C), infectious disease or musculoskeletal injury are not allowed to participate in the training session. If a participant reports any discomfort or incidence of injury during the intervention period, safety and further program continuation for the participant will be discussed in the research group, and in consultation with the

participant and the responsible physician. Investigators and responsible oncologists have the rights to exclude a participant from the study at any given time, if it is considered unsafe for the participant to continue. Data on adverse events will be collected systematically every week, and participants will further be informed to contact the primary investigator immediately if injuries or other adverse events related to the intervention program are required.

Publication of study results

Potentially positive, negative, or inconclusive results from the LOHAS-BCS study will be published in relevant international peer-reviewed journals. Co-authorship for the upcoming publications will be offered to members of the research group or other collaborators based on to the present work, and in accordance with the Vancouver Convention drawn up by the International Committee of Medical Journal Editors (ICMJE).

Finance for the study

The initiators of the LOHAS-BCS study are Jae Seung Chang, PhD. and In Deok Kong, MD, PhD. at Department of Physiology, Yonsei University Wonju College of Medicine. The LOHAS-BCS study is financially supported by a grant from the National Research Foundation of Korea funded by the Ministry of Science, ICT & Future Planning (NRF-2017R1A5A2015369) JSC is involved with. The support from the grant covers the cost of kits, reagents, materials and facilities, the partial salary of analyst for biomarker analyses. Financial support from other funding organizations will be applied, to cover expenses related to the LOHAS-BCS study. The Scientific Ethics Review Committee of the institute concerned and research members in the study will be informed about any further grants.

REFERENCES

1. Binkley JM *et al.*, Patient perspectives on breast cancer treatment side effects and the prospective surveillance model for physical rehabilitation for women with breast cancer. *Cancer* **118**, 2207-2216 (2012).
2. Loprinzi PD, Cardinal BJ, Effects of physical activity on common side effects of breast cancer treatment. *Breast Cancer* **19**, 4-10 (2012).
3. Knols R *et al.*, Physical exercise in cancer patients during and after medical treatment: a systematic review of randomized and controlled clinical trials. *J Clin Oncol* **23**, 3830-3842 (2005).
4. M. L. McNeely *et al.*, Effects of exercise on breast cancer patients and survivors: a systematic review and meta-analysis. *CMAJ* **175**, 34-41 (2006).

5 Nusse R, Clevers H. Wnt/beta-Catenin Signaling, Disease, and Emerging Therapeutic Modalities. *Cell* **169**, 985-99 (2017).

6. Polakis P, Wnt signaling in cancer. *Cold Spring Harb Perspect Biol* **4**, (2012).
7. Ghosh N, Hossain U, Mandal A, Sil PC. The Wnt signaling pathway: a potential therapeutic target against cancer. *Ann N Y Acad Sci* **1443**, 54-74 (2019).
8. Krishnamurthy N, Kurzrock R. Targeting the Wnt/beta-catenin pathway in cancer: Update on effectors and inhibitors. *Cancer Treat Rev* **62**, 50-60 (2018).
9. Clevers H, Nusse R. Wnt/beta-catenin signaling and disease. *Cell* **149**, 1192-205 (2012).
10. Nelson WJ, Nusse R. Convergence of Wnt, beta-catenin, and cadherin pathways. *Science* **303**, 1483-7 (2004).
11. MacDonald BT, Tamai K *et al*., Wnt/beta-catenin signaling: components, mechanisms, and diseases. Dev *Cell* **17**, 9-26 (2009).
12. Berschneider B, Konigshoff M. WNT1 inducible signaling pathway protein 1 (WISP1): a novel mediator linking development and disease. *Int J Biochem Cell Biol* **43,** 306-9 (2011).
13. Gurbuz I, Chiquet-Ehrismann R. CCN4/WISP1 (WNT1 inducible signaling pathway protein 1): a focus on its role in cancer. *Int J Biochem Cell Biol* **62**, 142-6 (2015)
14. Chiang KC *et al*., WNT-1 inducible signaling pathway protein-1 enhances growth and tumorigenesis in human breast cancer. *Sci Rep* **5**, 8686 (2015).
15. Tacke C *et al.*, Assessment of circulating Wnt1 inducible signalling pathway protein 1 (WISP-1)/CCN4 as a novel biomarker of obesity. *J Cell Commun Signal* **12**, 539-548 (2018).
16. Horbelt T *et al*., The novel adipokine WISP1 associates with insulin resistance and impairs insulin action in human myotubes and mouse hepatocytes. *Diabetologia* **61,** 2054-2065 (2018).
17. Murahovschi V *et al*., WISP1 is a novel adipokine linked to inflammation in obesity. *Diabetes* **64,** 856-66 (2015).
18. Cust AE *et al*., The influence of overweight and insulin resistance on breast cancer risk and tumour stage at diagnosis: a prospective study. *Breast Cancer Res Treat* **113**, 567-76 (2009).
19. Mendonca FM *et al.*, Metabolic syndrome and risk of cancer: which link? *Metabolism* **64**, 182-9 (2015).
20. Carmichael AR. Obesity and prognosis of breast cancer. *Obes Rev***7**, 333-40 (2006).

21 Sahin Ersoy G et al., WISP1 is a novel adipokine linked to metabolic parameters in gestational diabetes mellitus. *J Matern Fetal Neonatal Med* **30**, 942-946 (2017).

22. Murahovschi V et al., WISP1 is a novel adipokine linked to inflammation in obesity. *Diabetes* 64, 856-866 (2015).
